# Herausforderungen der ambulanten chirurgischen Versorgung

**DOI:** 10.1007/s00104-025-02263-6

**Published:** 2025-03-05

**Authors:** Johannes Klose, Claudia Rudroff, Lars Fischer, Artur Rebelo, Jörg Kleeff, Ralf Michael Wilke

**Affiliations:** 1 Klinik für Viszerale, Gefäß- und Endokrine Chirurgie, Universitätsmedizin Halle (Saale), Martin-Luther-Universität Halle-Wittenberg, Ernst-Grube-Str. 40, 06120 Halle (Saale), Deutschland; 2 Klinik für Viszeralchirurgie und funktionelle Chirurgie des unteren Gastrointestinaltrakts, Evangelisches Klinikum Köln Weyetal, Köln Weyetal, Deutschland; 3 Klinik für Allgemein- und Viszeralchirurgie, Klinikum Mittelbaden, Bühl, Mittelbaden Deutschland; 4 Klinik für Allgemein‑, Viszeral- und Gefäßchirurgie, BG-Klinikum Bergmannstrost, Halle (Saale), Deutschland; 5 Klinik für Allgemein- und Viszeralchirurgie, Diakoniekrankenhaus Halle (Saale), Halle (Saale), Deutschland; 6 Klinik für Allgemein‑, Viszeralchirurgie und Robotische Chirurgie, Asklepios Klinik Weissenfels, Weissenfels, Deutschland

**Keywords:** Ambulante Versorgung, Mindestmengen, EBM, Hybrid-DRG, AOP, Outpatient services, Minimum quantity, Unified assessment measure, Hybrid diagnosis-related groups, Outpatient surgical procedures

## Abstract

**Hintergrund:**

Der Anteil ambulanter und teilstationärer Operationen nimmt innerhalb der chirurgischen Leistungserbringung weiter zu. Zuletzt wurde der Katalog ambulant durchführbarer Operationen aktualisiert und eine sektorenübergreifende Vergütung eingeführt. Die Voraussetzungen in den chirurgischen Kliniken für eine vermehrte ambulante Leistungserbringung sind jedoch sehr heterogen.

**Ziele:**

Ziel dieser Arbeit ist, den aktuellen Stand und die Herausforderungen der ambulanten Versorgung in der Allgemein- und Viszeralchirurgie in Deutschland zu eruieren.

**Material und Methoden:**

Es wurde ein Fragebogen erstellt, in dem in 26 Fragen der Istzustand der ambulanten chirurgischen Versorgungsstruktur und die Möglichkeit der chirurgischen Ausbildung unter ambulanten Bedingungen abgefragt wurde. Dieser Fragebogen wurde digital über die Deutsche Gesellschaft für Allgemein- und Viszeralchirurgie sowie den Konvent der leitenden Krankenhauschirurginnen und -chirurgen versandt und die ausgefüllten Fragebögen ausgewertet.

**Ergebnisse:**

Teilgenommen haben 204 Chefärzte. In über 54,4 % leiten diese Grund- und Regelversorger, gefolgt von Schwerpunkt- und Maximalversorgern. In 95,5 % der Kliniken existieren ambulante Strukturen mit einem hohen Anteil medizinischer Versorgungszentren. Der Anteil der ambulanten Leistungserbringung soll ausgebaut werden, trotz noch nicht flächendeckend vorhandener Strukturen. Auch wenn kein dezidiertes Ausbildungskurrikulum vorhanden ist, werden ambulante Operationen auch von Assistenzärzten durchgeführt. Die aktuelle ambulante Versorgungslage wird von 70 % der Teilnehmenden als schlecht eingeschätzt; eine Verbesserung durch Einführung einer neuen sektorenübergreifenden Vergütung wird nicht erwartet.

**Diskussion:**

Innerhalb der Kliniklandschaft unterscheiden sich die Strukturen zur ambulanten chirurgischen Versorgung deutlich.

**Schlussfolgerung:**

Zwei Drittel der Teilnehmenden sehen ihre Klinik trotz der vorhandenen Infrastruktur nicht gut auf die Zunahme der ambulanten Leistungserbringung vorbereitet und bewerten die damit verbundene Vergütung als unzureichend.

**Graphic abstract:**

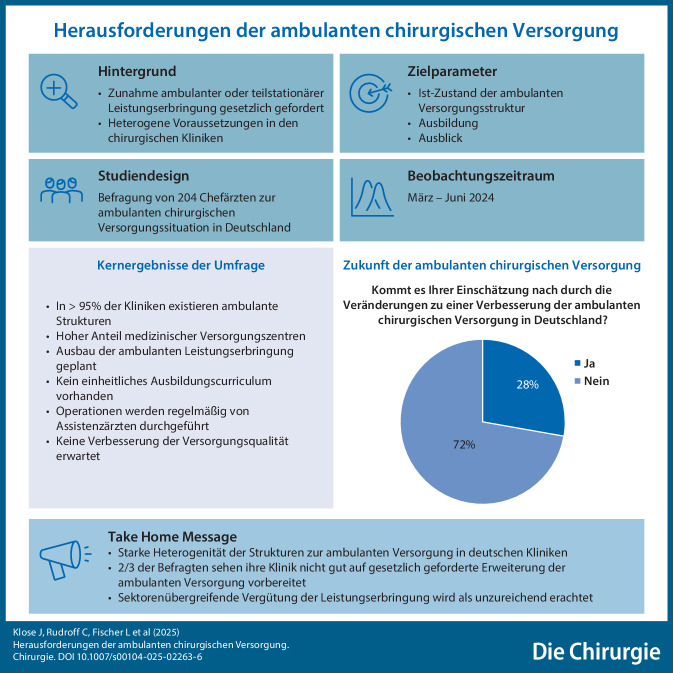

## Abstract (en)

**Background:**

In Germany the proportion of outpatient and day clinic surgery within the surgical service provision is still increasing. Recently, the catalogue of operations which can be carried out in outpatient surgery was revised and an intersectoral remuneration was introduced; however, the prerequisites in the surgical departments for an increased provision of outpatient services are very heterogeneous.

**Objective:**

The aim of this study was to analyze the current situation and challenges of outpatient care in general and visceral surgery in Germany.

**Material and methods:**

A questionnaire with 26 questions on the current situation of the treatment structure for outpatient surgery was constructed and the possibility for surgical training under outpatient conditions was surveyed. The questionnaire was digitally sent via the mailing list of the German Society for General and Visceral Surgery and the Convention of Senior Hospital Surgeons (*Konvent der leitenden Krankenhauschirurginnen und -chirurgen*) and the completed questionnaires were analyzed.

**Results:**

A total of 204 head physicians took part in the survey. In 54.4% the surgeons were in charge of basic care hospitals followed by specialized and maximum care hospitals. An outpatient structure was present in 95.5% of the hospitals with a high proportion of medical care centers. The majority of the surgeons planned to extend outpatient services despite the lack of comprehensive structures. Even if a structured education curriculum for junior doctors does not exist up to 70% of outpatient operations are performed by junior doctors. The current status of outpatient surgery care in Germany was evaluate as weak by 70% of the participants. An improvement due to the implementation of a new intersectoral remuneration is not expected.

**Discussion:**

Within the hospital landscape the structures for outpatient surgery show clear differences.

**Conclusion:**

Of the participants two thirds felt that despite the existing infrastructure their hospital was not well-prepared for the increased provision of outpatient services and assessed the associated remuneration as insufficient.

Der Anteil ambulanter und teilstationärer Operationen nimmt in Deutschland innerhalb der chirurgischen Leistungserbringung stetig zu. Für das Jahr 2024 wurde der Katalog ambulant durchführbarer Operationen aktualisiert und eine sektorenübergreifende Vergütung eingeführt. Damit verbunden war das Ziel, bestimmte chirurgische Leistungen vermehrt ambulant anzubieten und zu vergüten, damit personell und infrastrukturell teurere Eingriffe vermieden werden können. Das betrifft insbesondere die Hernienchirurgie. Das Ziel dieser Arbeit bestand darin, den aktuellen Stand und die Herausforderungen der ambulanten Versorgung in der Allgemein- und Viszeralchirurgie in Deutschland zu eruieren.

## Hintergrund und Fragestellung

Der Gesetzgeber fordert eine Steigerung der ambulanten operativen Leistungserbringung im deutschen Gesundheitssystem und hat in diesem Zug den Katalog ambulant durchführbarer Operationen (AOP-Katalog) zum 01.01.2024 aktualisiert [1, 2]. Basierend auf der Einigung der Kassenärztlichen Bundesvereinigung (KBV), der Deutschen Krankenhausgesellschaft (DKG) und des Spitzenverbands der gesetzlichen Krankenkassen (GKV) wurden 171 neue Operationen- und Prozedurenschlüssel (OPS) in den AOP-Katalog aufgenommen, der damit 3312 Prozeduren umfasst.

Erfahrungen aus dem europäischen Ausland zeigen, dass mit der Zunahme der ambulanten operativen Leistungserbringung bis zu 60 % der Klinikaufenthalte vermieden und die sektorenübergreifende Zusammenarbeit verbessert werden kann [3–6]. Gerade die COVID-Pandemie wurde im europäischen Ausland dazu genutzt, das Spektrum der ambulanten Operationsindikationen erfolgreich auszuweiten [7].

In Deutschland sind die berechnungsfähigen ambulanten und belegärztlichen Operationen im einheitlichen Bewertungsmaßstab (EBM) und weitere ambulant abrechenbare Leistungen für Krankenhäuser im AOP-Katalog aufgeführt. Seit dem 01.01.2024 regelt die Verordnung zur speziellen sektorengleichen Vergütung (Hybrid-DRG-V), dass definierte Leistungen niedergelassener Chirurgen und Krankenhäuser, unabhängig davon, ob sie ambulant oder stationär durchgeführt werden, nach einer einheitlichen Hybrid-DRG vergütet werden. Dadurch sollen vor allem operative Leistungen, die in Deutschland bislang stationär erbracht wurden, ambulant oder mit einer maximalen Verweildauer von einem Tag durchgeführt werden [8]. Dieser durch das Bundesgesundheitsministerium (BGM) definierte Katalog umfasst 34 OPS-Codes in 5 Leistungsbereichen, die für die Allgemein- und Viszeralchirurgie vor allem bestimmte Hernienoperationen, die Exzision eines Sinus pilonidalis und proktologische Eingriffe beinhalten [9].

Eine mögliche Konsequenz dieser Neustrukturierung der Vergütung operativer Leistungen soll zu einer vermehrten ambulanten Leistungserbringung führen.

Die aktuelle Infrastruktur zur ambulanten chirurgischen Versorgung variiert von Standort zu Standort erheblich und reicht von nicht vorhandenen Strukturen bis hin zu nahezu ideal ausgestatteten ambulanten Operationszentren. Strukturvorgaben hinsichtlich der personellen Ressourcen sowie der Aus- und Weiterbildung sind bislang ungeklärt.

Das Ziel dieser Arbeit bestand darin, mit einer Umfrage unter den Chefärzten der Krankenhäuser aller Versorgungsstufen den aktuellen Stand der ambulanten Versorgungssituation in der Allgemein- und Viszeralchirurgie in Deutschland zu erheben und zukünftige Haltungen und Perspektiven zu ermitteln.

## Studiendesign und Untersuchungsmethoden

Um den Istzustand der ambulanten Versorgungssituation in der Allgemein- und Viszeralchirurgie in Deutschland repräsentativ abbilden zu können, wurde ein Fragebogen mit insgesamt 26 Fragen erarbeitet. Der Fragebogen wurden anschließend mithilfe der REDCap-Software (Version 14.0.37) digitalisiert. Anschließend wurden Chefärzte einer Klinik der Allgemein- und Viszeralchirurgie elektronisch über die Verteilter der Deutschen Gesellschaft für Allgemein- und Viszeralchirurgie (DGAV) sowie den Konvent der leitenden Krankenhausärzte (KLK) kontaktiert und gebeten, an der Studie teilzunehmen. Dabei wurden Kliniken aller Versorgungsstufen in Deutschland (Grund- und Regelversorger, Schwerpunktversorger und Maximalversorger sowie die Universitätskliniken) miteingeschlossen. Die Studie wurde vom 25.03.2024 bis 30.06.2024 durchgeführt. Die Auswertung der Daten erfolgte nach Ende der Studie mithilfe der REDCap-Software. Dabei wurden alle eingegangenen und ausgefüllten Fragebögen ausgewertet.

## Ergebnisse

### Struktur und Charakteristika der teilnehmenden Kliniken

An der Umfrage beteiligten sich 204 Chefärzte. In Bezug auf die Versorgungsstufe waren 54,4 % der teilnehmenden Kliniken Grund- und Regelversorger (Stufe 1), 29,9 % Schwerpunktversorger (Stufe 2), 8,8 % Maximalversorger (Stufe 3) und 6,9 % aus den Universitätskliniken.

Die Anzahl der Krankenhaubetten der teilnehmenden Kliniken lag unter 250 bei einem guten Drittel (33,8 %), zwischen 250 und 399 Betten bei 25,9 % der Kliniken und zwischen 400 und 749 Betten bei 25,4 % der Kliniken. Über 750 bis 999 Betten verfügten 7 % Kliniken und 1000 Betten und mehr Betten hatten 8 % der teilnehmenden Kliniken.

Die Bettenzahl der rein viszeralchirurgischen Fachabteilungen einschließlich IMC- und ITS-Betten lag unter 25 Betten in 28,4 % Kliniken, zwischen 15 und 39 Betten in 40,6 % der Kliniken, zwischen 40 und 74 Betten in 26,4 % der Kliniken, zwischen 75 und 99 Betten in 3 % der Kliniken und über 100 Betten in 1,5 % der Kliniken.

Die Anzahl stationärer Fälle im Jahr 2023 lag bei über 75 % der teilnehmenden Kliniken über 1000 Fälle/Jahr (unter 750: 8,2 %; 750–1000: 15,8 %; 1000–1500: 33,2 %; über 1500: 42,9 %).

### Ambulante Operationen werden regelmäßig durchgeführt

In 95,5 % der Kliniken werden ambulante Operationen regelmäßig durchgeführt. In 18,5 % der Kliniken liegt der Anteil ambulanter Operationen über 20 %. In 22 % der Kliniken werden ca. 15 % der Eingriffe, in 45 % der Kliniken zwischen 5 und 10 % der Eingriffe und in 14,5 % der Kliniken weniger als 5 % der Eingriffe ambulant durchgeführt.

Der persönliche Anteil an ambulanten Operationen lag für 37,3 % der befragten Chefärzte unter 5 %, für 16,6 % der Befragten bei ca. 5 %, für 18,7 % der Teilnehmenden bei ca. 10 %, für 11,4 % der teilnehmenden Chefärzte bei ca. 15 % und für 16,1 % der Fälle bei 20 % und mehr.

### Organisation und Sektoren für die ambulante Chirurgie

Die Verteilung ambulanter Operationstermine wurde überwiegend von ärztlichen Mitarbeitern organisatorisch verantwortet (77,4 %). In 8,5 % der Fälle waren Mitarbeiter des Pflegedienstes und in 29,6 % der Fälle Mitarbeiter des Belegungsmanagements für die Organisation ambulanter Operationstermine verantwortlich.

Die Entscheidung, eine ambulante Operation durchzuführen, wurde in 7,4 % der Fälle vor der ersten Vorstellung getroffen, in 91,6 % der Fälle nach dem ersten Patientenkontakt und in 1 % am Operationstag selbst.

**Abb. 1:**
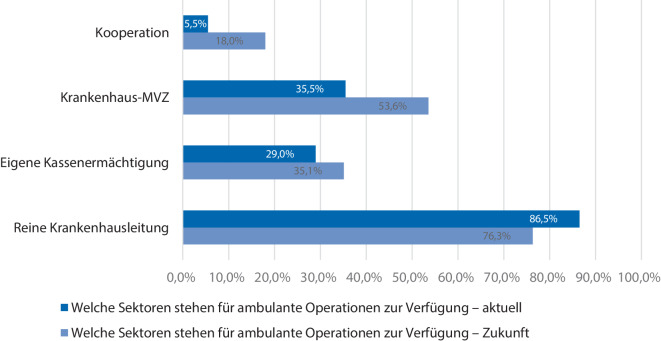
Aktuelle und geplante sektorale Organisation ambulanter Operationen

Die ambulanten Operationen werden in unterschiedlichen Sektoren organisiert. Es waren Mehrfachnennungen möglich. Die „ambulante Chirurgie“ ist reine Krankenhausleistung in 86,5 % der Fälle, erfolgt über eine eigene Kassenermächtigung in 29 % der Fälle, über ein Krankenhaus-MVZ in 35,5 % und in Form einer Kooperation mit anderen Krankenhäusern oder anderen Trägern in 5,5 % der Fälle (Abb. 1).

Zukünftig soll die ambulante Chirurgie bei den befragten Chefärzten in folgenden Sektoren organisatorisch strukturiert werden: als Krankenhausleistung in 76,3 % der Fälle, über eine eigene Kassenermächtigung in 35,1 % der Fälle, als eigenes Krankenhaus-MVZ in 53,6 % der Fälle und in Kooperation mit anderen Krankenhäusern oder anderen Trägern in 18 % der Fälle (Abb. 1).

Die räumliche Infrastruktur für die ambulante Versorgung verteilt sich aktuell auf einen eigenständigen Bereich innerhalb des Krankenhauses einschließlich eigener Station und OP in 34,7 % der Kliniken, in einer Tagesklinik in 10,1 % der Kliniken, als rein ambulanter OP in 21,6 % der Kliniken, als eigenständiger Bereich außerhalb des Krankenhauses in 12,6 % der Fälle und als rein stationäre Struktur in 36,7 % der Kliniken.

**Abb. 2:**
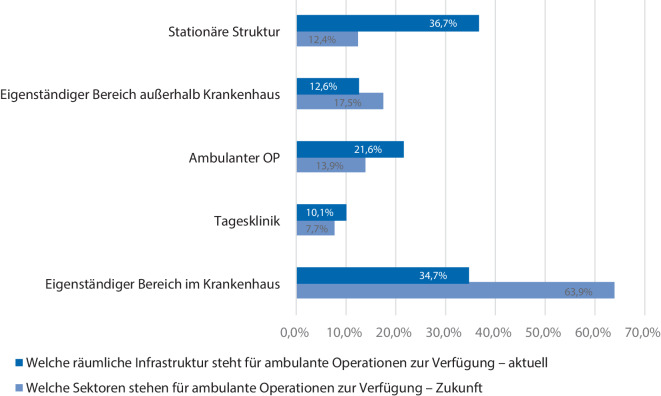
Aktuelle und geplante räumliche Infrastruktur ambulanter Operationen

Die räumliche Infrastruktur soll in den Kliniken zukünftig ausgebaut werden (Abb. 2). Ein eigenständiger Bereich innerhalb des Krankenhauses inklusive Station und OP soll in 63,9 % der Kliniken etabliert werden, in 7,7 % der Kliniken soll eine Tagesklinik eingerichtet werden, ein rein ambulanter OP soll in 13,9 % der Fälle genutzt werden, ein eigenständiger Bereich außerhalb des Krankenhauses soll in 17,5 % der Fälle zur Verfügung stehen und das Nutzen rein stationärer Strukturen soll in 12,4 % der Kliniken weitergeführt werden (Abb. 2).

Insgesamt 65,3 % der teilnehmenden Chefärzte planen, in Zukunft vermehrt eine Infrastruktur für ambulante Chirurgie zu nutzen. 23,1 % der Kollegen wollen gleichbleibend weiterarbeiten, und 11,6 % der Befragten möchten zukünftig weniger ambulante Chirurgie in der vorhandenen Infrastruktur im Krankenhaus durchführen.

### Personalstruktur

Aktuell gibt es in 28,9 % der Kliniken eigens für ambulante Operationen eingeteiltes Personal; 71,1 % der Kliniken nutzen das Personal sowohl für ambulantes Operieren als auch für das stationäre Operationsprogramm.

Insgesamt 45,5 % der befragten Chefärzte setzen überwiegend weniger als 2 % ihrer ärztlichen Mitarbeiter in der ambulanten Chirurgie ein. In 19,9 % der Kliniken werden zwischen 5 und 10 % der ärztlichen Mitarbeiter einer Abteilung für die ambulante Chirurgie eingesetzt. In 9,7 % der Kliniken werden mehr als 20 % der ärztlichen Mitarbeiter für ambulante Operationen abgestellt.

Die ambulanten Operationen wurden in den Kliniken zu 82,3 % von den Chefärzten durchgeführt. Oberärzte wurden in 92,4 % der Fälle, Fachärzte in 82,3 % und Assistenzärzte in 56,1 % der Fälle eingesetzt.

In 61,2 % der Kliniken werden die Eingriffe häufig von Assistenzärzten assistiert, in 30,3 % Kliniken ist dies selten der Fall, und in 8,5 % Kliniken werden gar keine Ausbildungseingriffe durchgeführt.

**Abb. 3:**
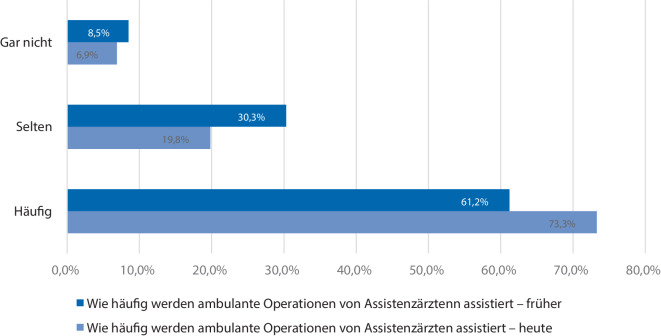
Häufigkeit der von Assistenzärzten durchgeführten ambulanten Operationen

Diese Zahlen unterscheiden sich von der früheren Praxis, wie das Balkendiagramm in Abb. 3 verdeutlicht. In 11 % Kliniken existiert ein strukturiertes spezielles Ausbildungskonzept für die ambulanten Operationen.

In den Kliniken, die bereits eigens eingeteiltes Personal für die ambulante Chirurgie einsetzen, ist dieses in 79,3 % der Fälle überwiegend fest eingeteilt und in 22,4 % der Fälle rotierend. In der überwiegenden Mehrzahl der Fälle wird auch internes Personal eingesetzt (90,9 %). 18,2 % der Kliniken nutzen externes Personal. Falls internes Personal für ambulante Chirurgie eingesetzt wird, macht das in 70,5 % der Kliniken weniger als 5 % des gesamten Personalbudgets der jeweiligen Abteilung aus.

**Abb. 4:**
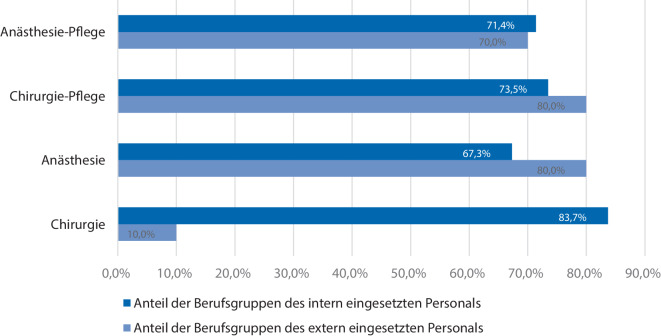
Verteilung der Berufsgruppen des intern bzw. extern eingesetzten Personals bei ambulanten Operationen

Die Verteilung der Fachgruppen ärztlicher Dienst Chirurgie, ärztlicher Dienst Anästhesie, Pflegedienst Anästhesie und Pflegedienst Chirurgie ist im Falle des intern eingeteilten Personals gleichmäßig verteilt (Abb. 4).

Beim Einsatz externen Personals wurde in 10 % der Fälle ein externer Chirurg eingesetzt. Dagegen waren externe Anästhesisten (80 %), externe Pflegekräfte der Anästhesie (70 %) und externe Pflegekräfte der Chirurgie (80 %) gleichverteilt (Abb. 4).

### Breite Vielfalt bei der Art der ambulanten Eingriffe

**Abb. 5:**
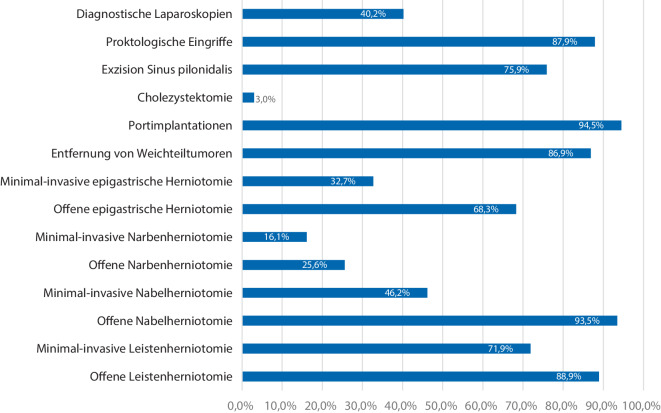
Ambulantes Operationsspektrum (Mehrfachnennungen möglich)

Die Art der ambulanten Eingriffe unterscheidet sich zwischen den verschiedenen Kliniken. Die Mehrzahl der befragten Kliniken (57,3 %) führen prinzipiell alle im AOP-Katalog aufgeführten oder laut Hybrid-DRG-Verordnung vorgesehenen Leistungen ambulant durch. 38,2 % der Kliniken führen diese Leistungen teilweise oder selektiv durch, 3,5 % der Kliniken führen Eingriffe über diese Kategorien hinausgehend durch und 1 % der Kliniken führen keine ambulanten Eingriffe nach oben genannten Kategorien durch. Die Art der Eingriffe verteilt sich wie in Abb. 5 dargestellt.

### Negative Einschätzung der Umfrageteilnehmer

Die Mehrheit der befragten Chefärzte planen eine Erweiterung des Angebots zur ambulanten Chirurgie.

Dabei sehen sich 26,4 % der Befragten aktuell gut vorbereitet auf die Anforderungen zur ambulanten Leistungserbringung, wohin gegen 73,6 % der Kollegen dies negativ bewerten.

**Abb. 6:**
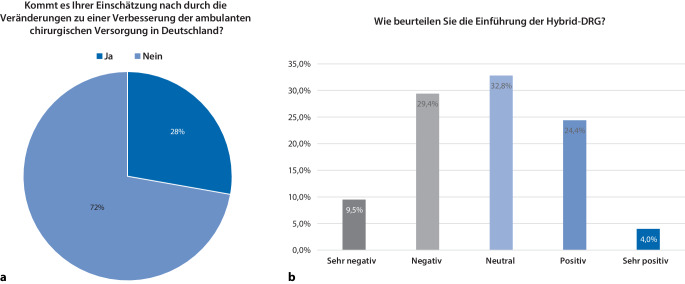
**a** Verbesserungspotenzial der Veränderungen zur ambulanten Versorgung und** b** Beurteilung der Einführung der Hybrid-DRG

Insgesamt 71,8 % der Befragten schätzen die Veränderungen als ungeeignet in Bezug auf eine Verbesserung der chirurgischen Versorgungsqualität im ambulanten Sektor ein (Abb. 6a).

Die Einführung der Hybrid-DRGs wird negativ (29,4 %) oder sehr negativ (9,5 %) bewertet; 33 % der teilnehmenden Chefärzte antworteten neutral, wohingegen 24,4 % der Umfrageteilnehmer die Veränderungen positiv oder sehr positiv (4 %) einschätzten. Diese Ergebnisse sind in Abb. 6b zusammenfassend dargestellt.

## Diskussion

Der Anteil der ambulanten Leistungserbringung in der Allgemein- und Viszeralchirurgie in Deutschland soll nach Wunsch des Gesetzgebers zunehmen – unter anderem, um personelle, räumliche und finanzielle Ressourcen zu schonen. Im Vergleich zum europäischen Ausland hinkt die ambulante Versorgung von Patienten in Deutschland insgesamt und in der Allgemein- und Viszeralchirurgie im Besonderen deutlich hinterher. So werden in Dänemark und dem Vereinigten Königreich bereits aktuell bis zu 80 % der Leistenherniotomien und fast 50 % der Cholezystektomien ambulant durchgeführt und dies ohne erhöhte Morbidität im internationalen Vergleich. In Österreich müssen die Kliniken ambulante Operationsquoten erfüllen, die an einen festgelegten Prozedurenkatalog gekoppelt sind [4, 10–16].

Um die Pläne der deutschen Gesundheitspolitik umzusetzen, wurde der AOP-Katalog überarbeitet und erweitert. Ab dem 01.01.2024 wurde mit der Einführung der Hybrid-DRG die sektorengleiche Vergütung der Leistungserbringung im Krankenhaus und für niedergelassene Chirurgen eingeführt und als ein weiterer Anreiz zur ambulanten operativen Leistungserbringung gesetzt.

Diese repräsentative Umfrage unter mehr als 200 chirurgischen Chefärzten von Krankenhäusern aller Versorgungsstufen in Deutschland, der überwiegende Teil aus Kliniken der Versorgungsstufen 1 und 2 und maximal 400 Betten, mit durchschnittlich über 1000 Behandlungsfällen pro Jahr und über 75 % stationären Fälle, spiegelt die Krankenhauslandschaft mit der für Deutschland spezifischen Heterogenität wider. Gerade in Krankenhäusern dieser Versorgungsstufen wurde durch Einführung der Hybridverordnung mit erlösrelevanten Einbußen gerechnet, die wiederum einen Einfluss auf zukünftige Personalstrukturen haben können. So beträgt etwa an einem beliebigen Grund- und Regelversorger der Anteil der Leistenhernienversorgung 20 % des Gesamtvolumens stationärer Fälle. Durch die bisherige a‑DRG-Vergütung und das Pflegeentgeld konnten diese Leistungen gewinnbringend angeboten werden. Durch die Einführung der Hybrid-DRG besteht nun eine Kürzung des Entgeltes von 30 %, sodass das bisherige Geschäftsmodell defizitär ist. Diese Leistungen sollten nun ambulant erfolgen, um wieder profitabel zu werden. Gleichzeitig führt dies wiederrum zu einem Verlust von stationären Fällen und Bewertungsrelationspunkten, sodass Bettenschließungen und zwangsläufig Reduktion des ärztlichen und pflegerischen Personalschlüssel resultieren [17].

Von den Befragten fühlten sich zwei Drittel auf die zunehmend geforderte Mehrerbringung ambulanter chirurgischer Leistungen nicht gut vorbereitet und stehen der neuen Form der Leistungsvergütung kritisch gegenüber. Das hat mitunter organisatorische Gründe, denn viele der von der Politik geplanten ambulanten Operationen werden aktuell noch überwiegend unter stationären Bedingungen durchgeführt. Dabei geht ein Großteil der erwarteten positiven Effekte verloren. Bei der notwendigen Etablierung eigenständiger ambulanter Strukturen fürchtet ein Großteil der Kliniken erhebliche Investitionen. Der geplante Anteil an MVZs zur ambulanten chirurgischen Leistungserbringung ist mit über 50 % recht hoch und soll perspektivisch noch erweitert werden. Potenziell kann hier eine Entlastung des stationären Versorgungssektors im Krankenhaus erreicht und ein kosteneffizientes Arbeiten durch eine prozessoptimierte Vor- und Nachbetreuung der Patienten gelingen, das in der klassischen Struktur eines Krankenhauses so nicht möglich wäre.

Die Mehrzahl der Kliniken führt gemäß dieser Befragung regelmäßig ambulante Operationen durch. Der Anteil der Kliniken, die mehr als 20 % ambulante Operationen durchführen, liegt aber nur bei 18,5 %, während der europäische Standard bei 20 % ambulanten Operationen insgesamt liegt [5]. Persönlich sind die befragten Chefärzte in der Regel nur bei maximal 5 % der ambulanten Operationen beteiligt.

Auch die Personalstruktur ist in den meisten Kliniken noch nicht geklärt. Die überwiegende Zahl der Kliniken setzt für das „ambulante Operieren“ das gleiche Personal ein, wie für die stationäre Versorgung und zwar überwiegend internes Personal. Beim Einsatz externen Personals kommt nur in einem Fall ein externer Chirurg zum Einsatz. Somit scheint die chirurgische Qualifikation eine, wenn nicht sogar die Schlüsselqualifikation für den Bereich „ambulantes Operieren“ zu sein.

Innerhalb des ärztlichen Dienstes setzen die befragten Chefärzte durchschnittlich maximal 2 % ihres ärztlichen Personals in der ambulanten Chirurgie ein. Dabei kommen mehrheitlich Ober- und Fachärzte zum Einsatz. Assistenzärzte werden zwar zu den Eingriffen hinzugezogen, aber weniger ausgebildet als im stationären Bereich. Im Vergleich zu der bislang erfolgten Einteilung zeigt sich aber ein Trend, dass Assistenzärzte häufiger für ambulante Operationen eingeteilt werden. Jedoch existiert nur in etwas mehr als 10 % ein dezidiertes Ausbildungskonzept für das ambulante Operieren. Dies ist ein Aspekt, der im Hinblick auf die zukünftige Strukturentwicklung angegangen werden muss. Hier muss in Zukunft über ein dezidiertes, von der Fachgesellschaft miterarbeitetes Ausbildungskurrikulum diskutiert werden, damit einerseits die zu erbringende ambulante chirurgische Leistung rasch und sicher erfolgt, andererseits aber auch die angehenden Chirurgen im Rahmen ihrer Facharztausbildung genau diese Eingriffe lernen. Bedingt durch den steigenden Kostendruck auf die Kliniken kann es zu einer Verschlechterung der Ausbildung des chirurgischen Nachwuchses ohne dezidiertes Ausbildungskonzept kommen. Dabei könnte die hohe Fallzahl jedoch auch genutzt werden, um schnell eine Routine in der Durchführung der Eingriffe für weniger erfahrene Chirurgen zu ermöglichen.

Die Art der ambulanten Eingriffe unterscheidet sich von Klinik zu Klinik. Auch wenn die Mehrzahl der befragten Kliniken überwiegend die im AOP-Katalog bzw. in den Hybrid-DRGs geforderten Eingriffe durchführt, variiert die tatsächliche Umsetzung erheblich. Grundsätzlich sind aber fast alle Chefärzte der Meinung, dass sie in ihrer Klinik zukünftig mehr ambulante Operationen durchführen werden als bisher. Es zeigt sich, dass in Deutschland die ambulante Cholezystektomie unterrepräsentiert ist. In nur 8 % der teilnehmenden Kliniken werden regelmäßig ambulante Entfernungen der Gallenblase durchgeführt. Im europäischen Ausland hingegen erfolgt die Hälfte aller Cholezystektomien als ambulanter Eingriff [14, 15].

Bei deutlichem Interesse an einer Ausweitung der ambulanten Versorgungsangebote, insbesondere durch den Ausbau eigenständiger Bereiche innerhalb und außerhalb der Kliniken, sieht sich dennoch ein erheblicher Anteil der befragten Kliniken nicht ausreichend vorbereitet. Insbesondere werden Defizite in der personellen und infrastrukturellen Ausstattung beklagt. Diese Studie ermöglicht es jedoch nicht, diese bestehenden Defizite in der ambulanten chirurgischen Versorgung trotz jahrelanger Bestrebungen des Gesetzgebers zur Stärkung des ambulanten Sektors zu erklären. Auch die Einführung der Hybrid-DRGs wird sehr unterschiedlich bewertet. Auffällig ist die Diskrepanz zwischen der überwiegend großen Teilnahme an der ambulanten Leistungserbringung und der überwiegend kritischen Bewertung des Ambulantisierungsprozesses – ein unterstützendes Begleiten des Prozesses wäre hier sicher wichtig und sollte im Vordergrund stehen. Die Tatsache, dass das „ambulante Operieren“ international mittlerweile fest etabliert ist, ohne dass es zu Einbußen in der Patientenversorgung gekommen ist, sollte ermutigen, die weiteren Schritte in diesem Bereich zu initiieren und proaktiv zu begleiten [18].

## Fazit für die Praxis

Ambulante Operationen werden einen immer größeren Stellenwert in der chirurgischen Versorgung in Deutschland einnehmen. Die von den chirurgischen Chefärzten geäußerte Skepsis gegenüber den damit verbundenen Regularien und Vergütungen kann nur durch gesundheitspolitische Unterstützung entkräftet werden. Von den chirurgischen Fachgesellschaften wäre die Forderung nach einer kostendeckenden Vergütung ambulanter Operationen im Krankenhaussetting genauso wie die Mitentwicklung eines strukturierten chirurgischen Ausbildungskurrikulums wünschenswert.

## Data Availability

Die erhobenen Datensätze können auf begründete Anfrage in anonymisierter Form beim korrespondierenden Autor angefordert werden. Die Daten befinden sich auf einem Datenspeicher der Klinik für Viszerale, Gefäß- und Endokrine Chirurgie der Universitätsmedizin Halle (Saale).
